# Gene expression variation between distinct areas of breast cancer measured from paraffin-embedded tissue cores

**DOI:** 10.1186/1471-2407-8-343

**Published:** 2008-11-25

**Authors:** Martina Schobesberger, Anna Baltzer, Andrea Oberli, Andreas Kappeler, Mathias Gugger, Hana Burger, Rolf Jaggi

**Affiliations:** 1 Department of Clinical Research, University of Bern, Murtenstrasse 35, CH-3010 Bern, Switzerland; 2 Institute of Pathology, University of Bern, Murtenstrasse 31, CH-3010 Bern, Switzerland

## Abstract

**Background:**

Diagnosis and prognosis in breast cancer are mainly based on histology and immunohistochemistry of formalin-fixed, paraffin-embedded (FFPE) material. Recently, gene expression analysis was shown to elucidate the biological variance between tumors and molecular markers were identified that led to new classification systems that provided better prognostic and predictive parameters. Archived FFPE samples represent an ideal source of tissue for translational research, as millions of tissue blocks exist from routine diagnostics and from clinical studies. These should be exploited to provide clinicians with more accurate prognostic and predictive information. Unfortunately, RNA derived from FFPE material is partially degraded and chemically modified and reliable gene expression measurement has only become successful after implementing novel and optimized procedures for RNA isolation, demodification and detection.

**Methods:**

In this study we used tissue cylinders as known from the construction of tissue microarrays. RNA was isolated with a robust protocol recently developed for RNA derived from FFPE material. Gene expression was measured by quantitative reverse transcription PCR.

**Results:**

Sixteen tissue blocks from 7 patients diagnosed with multiple histological subtypes of breast cancer were available for this study. After verification of appropriate localization, sufficient RNA yield and quality, 30 tissue cores were available for gene expression measurement on TaqMan^® ^Low Density Arrays (16 invasive ductal carcinoma (IDC), 8 ductal carcinoma in situ (DCIS) and 6 normal tissue), and 14 tissue cores were lost. Gene expression values were used to calculate scores representing the proliferation status (PRO), the estrogen receptor status and the HER2 status. The PRO scores measured from entire sections were similar to PRO scores determined from IDC tissue cores. Scores determined from normal tissue cores consistently revealed lower PRO scores than cores derived from IDC or DCIS of the same block or from different blocks of the same patient.

**Conclusion:**

We have developed optimized protocols for RNA isolation from histologically distinct areas. RNA prepared from FFPE tissue cores is suitable for gene expression measurement by quantitative PCR. Distinct molecular scores could be determined from different cores of the same tumor specimen.

## Background

Diagnosis and prognosis of breast cancer are still mainly based on clinical, histological and immunohistochemical parameters, which are at best semi-quantitative [1,2]. Recently, molecular characterization of breast cancer has greatly increased the understanding of biological pathways that are altered during neoplastic transformation. Molecular markers have a great impact on elucidating the biological variance within tumors, they allow new and more accurate classifications and they have the potential to improve diagnosis, estimation of prognosis and treatment decisions in individual patients [3-6].

Most gene expression studies are based on fresh frozen material which, in most instances, is not readily available, as surgical samples are usually fixed in formalin. Unfortunately, RNA derived from formalin-fixed, paraffin-embedded (FFPE) material is considerably fragmented and chemically modified, often impairing gene expression measurement using standard procedures. We developed a simple and robust protocol for RNA isolation and partial de-modification from standard FFPE sections and documented that the isolated RNA is suitable for gene expression measurement by quantitative reverse transcription PCR (QRT-PCR) [7,8]. However, RNA isolated from tissue sections may not be representative for a tumor as the proportion of normal tissue in a section may be significant. To circumvent this problem, we used tissue cores as prepared for the construction of tissue microarrays (TMA). TMAs allow to analyze hundreds of archival tissue samples simultaneously [9]. For a TMA, individual cores are punched from representative areas of a large series of FFPE tissue blocks and re-assembled on a single recipient paraffin block. Sections from TMA blocks are processed by staining, immunohistochemistry or *in situ *hybridization like regular tissue sections, revealing results from up to 1000 individual tissue cores present on a single array [10,11].

We used such tissue cores as source of material for RNA isolation. In contrast to sections which represent all tissue types present in the block, tissue cores have the great advantage that they can be taken very precisely from the area of interest within an individual tissue block [12,13]. In the present study, tissue cores were taken from normal and cancerous tissue of the same block and gene expression was measured. We compared the level of expression of various genes between invasive ductal carcinoma (IDC), ductal carcinoma in situ (DCIS) and regions of histologically normal breast epithelium in single tissue blocks.

## Methods

### Tissue specimens and generation of tissue cores

**Figure 1:**
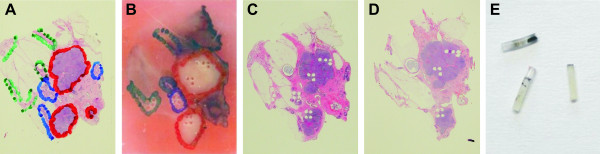
**FFPE material before and after punching**. Hematoxylin and eosin-stained tissue sections and paraffin block of patient 4 are shown before and after punching tissue cores. Areas of IDC (red), DCIS (blue) and normal tissue (green) were marked on the original tissue section (A) and on the corresponding paraffin block (B). Tissue cores (E) were taken with a tissue microarrayer instrument. Thereafter, sections were made from the remaining block and a control staining and RNA isolation were done at ~400 μm intervals through the block (C and D). The area containing DCIS was lost after ~400 μm and tissue cores contained fat instead of tumor tissue. It was known from previous experiments that very little RNA can be isolated from fat cells. Therefore, expression measurements in tissue cores containing fat besides tumor tissue are not compromised.

Breast cancer specimens from 7 patients diagnosed with ductal carcinoma were retrieved from the files of the Institute of Pathology, University of Bern. Tissue samples were fixed with 4% neutralized formalin and embedded in paraffin. Sections from each of a total of 16 tissue blocks (1 to 4 blocks per patient) were stained with hematoxylin and eosin and regions containing IDC, DCIS and normal breast epithelium were marked on each slide and the corresponding paraffin block (Fig. 1A and 1B). Three to 5 tissue cores were punched from each area of interest (1 to 5 areas in each block) using a manual tissue microarrayer (Beecher Instruments, Sun Prairie, WI, USA). In total, 3 to 5 tissue cores were taken from 44 individual areas. Cylinders had a diameter of 0.6 mm and the length of the cores varied between 1 and 3 mm resulting in approximately 0.3 to 1 mg tissue per core (Fig. 1E). After taking tissue cores, RNA was isolated from five 10 μm thick serial sections that were collected from each tissue block at several levels separated by 400 μm. Tissue was evaluated by hematoxylin and eosin staining of sections at each level and the localization of tissue cores was verified microscopically within tumor and normal tissue (Fig. 1C and 1D). Six tissue cores were excluded from further experiments because they did not represent tissue from the appropriate area of interest. Histopathological characteristics of each tumor, number of layers per block and number of areas punched and analyzed are summarized in Table 1. All patients gave written informed consent to use their material. The study was performed on the basis of an approval by the ethical committee of the Canton Bern, Switzerland.

**Table 1:** Breast tissue specimens used in the study

Patient No.	Histological grade	IHC				Block	No. of layers	No. of tissue cores^1^		
					
		ER	PgR	Her2	Ki-67 LI (%)			IDC	DCIS	N
		
1	3	+	+	-	70	1.1	2	3/3	1/1	1/1
2	2	+	+	-	n.d.	2.1	3	2/2	1/1	1/1
3	3	+	+	+	50	3.1	2	3/3	1/1	0/1^2^
4	2	+	-	+	30	4.1	3	3/3	1/1	1/1
						4.2	3		1/1	
						4.3	3			0/1^3^
5	2	+	+	+	5	5.1	3	2/2		
						5.2	2		1/1	0/1^3^
						5.3	1	0/1^4^	0/1^3^	0/1^4^
						5.4	2			1/1
6	3	-	-	+	80	6.1	3	1/2^2^	1/1	0/2^2^
						6.2	3		1/2^4^	
						6.3	3		0/1^4^	
						6.4	3			1/2^2^
7	2	+	+	-	10	7.1	3	2/3^4^		
						7.2	1			1/2^4^

IHC = immunohistochemistry; ER = estrogen receptor; PgR = progesteron receptor; Her2 = Her2/neu; Ki-67 LI = Ki-67 labeling index; N = normal tissue; n.d. = not determined; ^1 ^= number of tissue cores analyzed/total number of tissue cores punched; ^2 ^= tissue cores excluded from gene expression measurement due to insufficient RNA yield; ^3 ^= tissue cores excluded from gene expression measurement due to insufficient RNA quality; ^4 ^= tissue cores excluded from gene expression measurement due to inappropriate localization of the core.

### RNA isolation and expression analysis

Entire tissue cores or 5 tissue sections were de-paraffinized with Neo-Clear (Merck, Darmstadt, Germany), washed with ethanol and dried. RNA was isolated as described previously [8] using the following modifications: 500 μl of lysis buffer containing approximately 1 μg/μl Proteinase K (Roche Diagnostics, Mannheim, Germany) was used. Tissue was homogenized in a Mixer Mill at 20 Hz for 4 min and digested at 55°C for 1 hour. RNA was de-modified and purified on silica-based columns as described previously [8]. RNA quantity and quality were assessed on a ND-1000 spectrophotometer (NanoDrop Technologies, Wilmington, DE, USA) and on an Agilent 2100 Bioanalyzer (Agilent Technologies, Inc., Santa Clara, CA, USA). Five tissue cores were excluded from subsequent experiments due to insufficient RNA yield (less than 0.45 μg).

The quality of each RNA sample was further tested by QRT-PCR using TaqMan assays for GUSB, RPLP0 and UBB (Applied Biosystems, Foster City, CA, USA) (Table 2) and a One-Step QRT-PCR protocol according to the recommendations of the manufacturer (Invitrogen, Carlsbad, CA, USA). QRT-PCR was performed on an Applied Biosystems 7500 instrument in Fast mode (50°C for 15 min, 95°C for 2 min, followed by 45 cycles of 95°C for 3 sec and 60°C for 30 sec). Three RNA samples were excluded from further analyses because the mean Ct value of the 3 control genes was considerably higher than the mean of all other samples.

**Table 2:** Gene expression assays used for QRT-PCR

**Symbol**	**Gene description**	**Accession No.**	**Category**	**Amplicon Size**
GUSB	glucuronidase, beta	NM_000181.1	Control	81
RPLP0	ribosomal protein, large, P0	NM_053275.3 NM_001002.3	Control	105
UBB	ubiquitin B	NM_018955.2	Control	120
BCL2	B-cell CLL/lymphoma 2	NM_000633.2	ER	81
ESR1	estrogen receptor 1	NM_000125.2	ER	62
PGR	progesterone receptor	NM_000926.3	ER	118
SCUBE2	CEGP1, signal peptide, CUB domain, EGF-like 2	NM_020974.1	ER	64
ERBB2	v-erb-b2 erythroblastic leukemia viral oncogene homolog 2, neuro/glioblastoma derived oncogene homolog (avian)	NM_001005862.1 NM_004448.2	Her2	120
GRB7	growth factor receptor-bound protein 7	NM_005310.2	Her2	70
AURKA	STK15 aurora kinase A	NM_003600.2	Proliferation	85
BIRC5	baculoviral IAP repeat-containing 5 (survivin)	NM_001012271.1 NM_001168.2	Proliferation	93
CCNB1	cyclin B1	NM_031966.2	Proliferation	104
MKI67	antigen identified by monoclonal antibody Ki-67	NM_002417.3	Proliferation	131
MYBL2	v-myb myeloblastosis viral oncogene homolog (avian)-like 2	NM_002466.2	Proliferation	81

Shown are genes on the TLDA that were used for computing the PRO, ER and HER2 scores and the three control genes used for normalization [8].

For the remaining 30 RNA samples gene expression levels were measured for control genes and for genes associated with proliferation, estrogen receptor function and HER2 using TaqMan^® ^Low Density Arrays (TLDA; Applied Biosystems) on an Applied Biosystems 7900 HT instrument. A list of assays used in this study is given in Table 2. Individual reactions were carried out with 2 ng of total RNA using the following cycling conditions: 50°C for 15 min, 95°C for 2 min, followed by 40 cycles of 95°C for 10 sec and 60°C for 30 sec.

### Normalization of data

Raw data were recorded with the SDS software of the instruments. Delta cycle threshold (Ct) values were determined as the difference between the Ct of each test gene and the mean of RPLP0, UBB and GUSB (control genes). Scores representing the proliferation status (PRO), the estrogen receptor status (ER) and the HER2 status (HER2) were computed from 5 genes associated with proliferation (AURKA, MYBL2, CCNB1, MKI67 and BIRC5), 4 genes related to estrogen receptor function (ESR1, PGR, BCL2 and SCUBE2) and 2 genes related to HER2 (ERBB2 and GRB7). The procedure for calculating scores was described previously [8].

## Results and Discussion

Sixteen tissue blocks from 7 breast cancer patients diagnosed with multiple histological subtypes were available for this study. In total, 44 tissue cores were taken from areas representing 19 IDC, 11 DCIS and 14 areas containing normal tissue. After verification of appropriate localization, sufficient RNA yield and quality, 30 tissue cores were available for gene expression measurement on TLDAs (16 IDC, 8 DCIS and 6 normal tissue) (Table 1).

**Figure 2:**
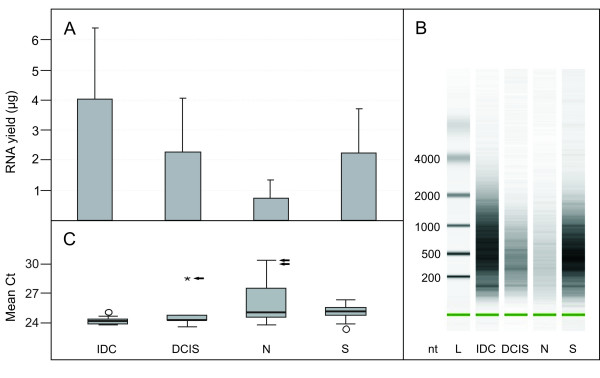
**Characterization of RNA**. RNA was isolated from tissue cores and from histological sections and RNA concentration was measured spectrophotometrically (A). RNA yield was converted to correspond to 3 tissue cores with an average length of 3 mm or to 1 tissue section (10 μm thick), respectively. Mean recoveries and corresponding standard deviations are shown for 17 IDC, 9 DCIS and 12 normal tissue cores (N) and for 40 tissue sections (S). Five RNAs were excluded from subsequent experiments because RNA yield was less than 0.45 μg. The quality of each RNA sample was tested on a Bioanalyzer. A representative, gel-like picture for each histological type is shown (B). RNA quality was further assessed by QRT-PCR and mean Cts of 3 control genes (GUSB, RPLP0 and UBB) are shown (C). One RNA sample from DCIS and 2 RNA samples from normal tissue were excluded from subsequent expression measurement because mean Ct values of control genes were more than one standard deviation higher than the mean of all Cts (arrows).

Total RNA was isolated from tissue cores and from sections as described in Methods and according to the previously established protocol [8]. RNA yield was assessed spectrophotometrically (Fig. 2A). For a better comparison of recoveries from individual samples, the RNA yield was converted to numbers corresponding to 3 tissue cores with an average length of 3 mm or to 1 tissue section (10 μm thick), respectively. The mean recovery from tissue cores was 4.0 μg (range 0.3 – 8.5) for IDC, 2.3 μg (0.8 – 6.2) for DCIS and 0.8 μg (0.2 – 2.2) for normal tissue. The corresponding mean yield per tissue section was 2.3 μg (range 0.3 – 6.3). The quality of each RNA was tested on a Bioanalyzer (Fig. 2B) and by measuring gene expression of 3 control genes (GUSB, RPLP0 and UBB) by QRT-PCR (Fig. 2C). Bioanalyzer results revealed fragment lengths between 100 and 2000 bp with mean fragment lengths around 500 bp which is typical for good quality RNA from FFPE material. The mean of all 3 control genes was Ct = 24.3 (range 23.8 – 25.2) for IDC, Ct = 24.8 (23.6 – 28.5) for DCIS and Ct = 26.0 (23.9 – 30.3) for normal tissue cores. Corresponding mean expression for tissue sections revealed Ct = 25.1 (range 23.4 – 26.3).

**Figure 3:**
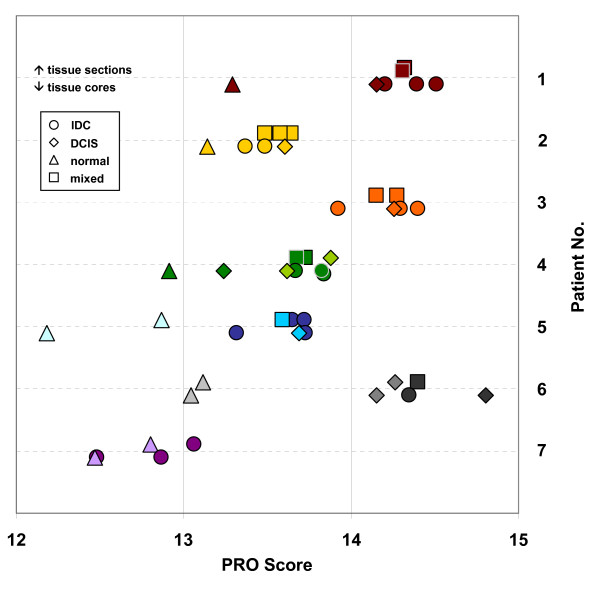
**PRO scores from tissue cores and from sections**. PRO scores were computed from tissue cores (symbols below dashed lines) and from sections taken at different levels of tissue blocks (symbols above dashed lines). Scores derived from IDC are represented by circles, DCIS by diamonds and normal tissue by triangles. Sections containing IDC, DCIS and/or normal tissue are shown as squares, sections containing only one tissue type are given with the same symbols as tissue cores. Cores and sections derived from the same tissue block are shown in the same color.

Gene expression levels were measured on TLDAs. Gene expression of each test gene was normalized using the mean of RPLP0, GUSB and UBB as reference. Putative control genes were previously compared by QRT-PCR with RNA of more than 80 matched samples of fresh frozen and archival FFPE material, which revealed that GUSB, RPLP0 and UBB are the 3 most stably expressed genes in breast cancer that can be measured reliably by QRT-PCR (Antonov et al., manuscript in preparation). Scores were computed from genes related to PRO, ER and HER2 status as described in Methods. Fig. 3 shows PRO scores computed from tissue cores (symbols below dashed lines) and from sections (symbols above dashed lines) for all 7 patients. Tissue cores and sections derived from the same block are shown in the same color. Tissue cores from IDC, DCIS and normal tissue are represented as circles, diamonds and triangles, respectively. Sections representing a mixture of more than one tumor type are depicted as squares. Replicate cores taken from histologically homogeneous tissue regions gave similar scores (tissue cores from patients 1–5) corroborating that the procedure for determining PRO scores is robust. Similar scores were also measured between tissue cores and sections in cases where blocks contained only one type of tumor or normal tissue (for example patient 6).

Blocks from several patients contained IDC, DCIS and normal tissue in the same paraffin block (patients 1, 2 and 4). In all these cases, the PRO scores measured from entire sections were similar to PRO scores determined from IDC tissue cores. This is most likely due to a higher RNA content in tumor cells than in normal breast tissue. Scores determined from normal tissue cores consistently revealed low PRO scores while PRO scores were higher for IDC or DCIS of the same block or from different blocks of the same patient (patients 1–6). Samples from patient 7 contained IDC which had a relatively low PRO score corresponding to cancers which have a more favorable prognosis than cancers which have high PRO scores (Antonov et al., manuscript in preparation). In all patients PRO scores were fairly similar for DCIS and IDC. A similar observation was reported previously by others [14,15] who compared atypical ductal hyperplasia, DCIS and IDC and showed high degrees of similarity at the level of gene expression between these pathological stages. Both studies were based on fresh frozen material.

Tissue cores used in this study had a length of about 3 mm. To investigate whether the tissue was histologically homogenous in the vertical dimension, 3 mm of each block were sectioned and every 400 μm one section was stained and 5 sections were taken for RNA isolation. In most cases morphology was similar at different levels (Fig. 1A, C and 1D). Similarly, RNA derived from each layer gave similar PRO scores (Fig. 3, blocks from patients 1 – 5, upper rows). This is in agreement with other studies, which showed by TMA and immunohistochemistry that data generated from tissue cores yield clinically significant and representative results [16,17]. The PRO score which is generated from proliferation-related genes was also compared to Ki-67 labeling index which was determined by immunohistochemistry from sections. The PRO scores determined from tissue cores of IDC and DCIS or from tissue sections in the 7 patients are in good agreement with Ki-67 labeling index (Table 1 and Fig. 3). Tissue cores were also evaluated with respect to ER and HER2 scores confirming that RNA from tissue cores is suitable for gene expression measurement (data not shown).

## Conclusion

The method presented here is suitable for FFPE tissue, it can be combined with traditional TMA studies without great additional effort allowing to match molecular markers with morphological, immunohistochemical and/or *in situ *analyses. Comparing stained tissue sections before and after taking tissue cores allows to control very precisely whether material was taken from the expected area and cores representing unwanted tissue regions can be excluded from further analysis. Sufficient RNA can be isolated from 3 to 5 tissue cores for multiple QRT-PCR analyses (at least 100 reactions) without the need for RNA amplification.

## Competing interests

The authors declare that they have no competing interests.

## Authors' contributions

MS and RJ organized the study, planned the experiments and wrote the manuscript. AO and MS carried out RNA isolations, quality controls and gene expression measurements. AB was responsible for preparation of tissue cores, sections and stainings. HB and MG selected the samples and carried out the histopathological assessment. AK was responsible for histology and diagnostic stainings. All authors read and approved the final manuscript.

## Pre-publication history

The pre-publication history for this paper can be accessed here:


